# A retrospective study of 3D-printed custom titanium prostheses for reconstruction of bone defects after resection of pelvic tumors: technical points and early results

**DOI:** 10.1186/s12893-025-03253-5

**Published:** 2025-10-31

**Authors:** Tiying Wang, Xiang Ma, Ya Zhang, Jing Li, Lei Han, Zia-ul-Haq Nabil, Yan Liu, Linhao Cai, Weiquan Wang, Zhou Huang, Zuozhang Yang

**Affiliations:** Bone and Soft Tissue Tumors Research Center of Yunnan Province, Department of Orthopaedics, The Third Affiliated Hospital of Kunming Medical University, Yunnan Cancer Hospital, Peking University Cancer Hospital Yunnan, Kunming, Yunnan, 650118 China

**Keywords:** 3D-printed, Customized pelvic prosthesis, Technology, Early results, Literature review

## Abstract

**Background:**

With the development of limb salvage technology for pelvic malignant tumor, 3D-printed custom prosthesis has become a main way to reconstruct pelvis after resection of pelvic malignant tumor. the purpose of this study is to explore the technical points of using 3D-printed custom titanium alloy prosthesis to reconstruct bone defect after resection of pelvic tumor, and to analyze the early curative effect, complications and prognosis, in order to provide clinical reference for the resection and reconstruction of pelvic malignant tumor.

**Methods:**

We reviewed 7 patients (4 males, 3 females; mean age 42 years, range 16–54) receiving 3D-printed titanium alloy prostheses for pelvic reconstruction following malignant tumor resection (June 2022-December 2023). Cases included 4 primary bone malignancies (2 chondrosarcomas, 2 osteosarcomas) and 3 secondary malignant tumors (endometrial, rectal, hepatocellular carcinomas). Enneking zones involved I + II/I + II + III/II + III (*n* = 4) and I + II + IV/I + II + III + IV (*n* = 3), with resections including wide (*n* = 3), marginal (*n* = 3), and intracapsular (*n* = 1). VAS and KPS assessed pain and physical strength preoperatively and at 1/3/6 months postoperatively. MSTS 93 evaluated limb function pre-/postoperatively. Pelvic stability was assessed via pre- and 6-month postoperative sacroiliac joint space measurements. Complications were documented during follow-up (mean 10.6 months, range 6-18.2), while overall survival, progression-free survival, and prognostic factors were analyzed using Kaplan-Meier/COX methods.

**Results:**

All 7 patients underwent successful procedures, comprising 5 standard semi-pelvic/iliac prosthesis implantations and 2 screw-connected semi-pelvic reconstructions. Significant improvements were observed in both pain and functional outcomes. The median preoperative VAS score was 7.0 (IQR 6.0-8.5), which decreased to 4.0 (3.5-5.0) at 1 month, 3.0 (2.0-4.5) at 3 months, and 2.5 (2.0-3.5) at 6 months (χ² = 9.46, *P* = 0.024), with all pairwise comparisons remaining significant after Bonferroni correction (*P* < 0.001). Simultaneously, median KPS scores improved from 60.0 (40.0–60.0) preoperatively to 60.0 (50.0–70.0) at 1 month, 70.0 (60.0–80.0) at 3 months, and 80.0 (70.0–80.0) at 6 months (χ² = 13.36, *P* = 0.004), with all inter-timepoint comparisons also statistically significant (*P* < 0.001). The mean MSTS 93 score was 11.00 ± 4.20 preoperatively and significantly improved to 23.86 ± 4.06 at 3 months postoperatively (*P* < 0.001). A significant difference in the contralateral sacroiliac joint space was observed between preoperative and 6-month postoperative measurements in patients with Zone IV involvement (IV group) (*P* = 0.048). Complications included heterotopic ossification (4/7, 57.1%), pubic screw fracture (1/7, 14.3%), femoral neck dislocation (1/7, 14.3%), delayed wound healing (1/7, 14.3%), local recurrence (2/7, 42.8%), and distal metastasis (2/7, 42.8%). No deep infection, periprosthetic osteolysis, or implant loosening occurred. With a mean follow-up of 10.6 months (range: 6–18.2 months), one patient died due to tumor recurrence and metastasis. Kaplan–Meier analysis and Cox multivariate regression showed that gender, tumor type, extent of resection, and Zone IV involvement were not significant variables affecting progression-free survival.

**Conclusion:**

3D-printed custom titanium alloy prostheses exhibit favourable early efficacy, safety, and prognostic outcomes in the reconstruction of pelvic defects following tumor resection. Nevertheless, observed alterations in the contralateral unaffected sacroiliac joint space following Zone IV reconstruction have led to a hypothesis regarding potential instability risk.

**Supplementary Information:**

The online version contains supplementary material available at 10.1186/s12893-025-03253-5.

## Introduction

 The pelvis is a common site for malignant bone tumors, and pelvic tumor surgery is a great challenge for bone tumor specialists due to its deep location and complex anatomy, and pelvic tumors are often already large in size and adjacent to complex surrounding organs and blood vessels by the time of the first visit [1, 2]. Prior to the 1980 s, the usual surgical approach for pelvic malignancies was hemi-pelvic amputation, but in 1978 Enneking et al. [2] and Steel [1], among others, reported that partial hemi-pelvic resections with guaranteed surgical boundaries did not have a significantly higher local recurrence rate than hemi-pelvic amputations.

Subsequently, limb preservation techniques were developed and gradually replaced amputation as a standard of surgical treatment for pelvic tumors. Reconstruction of the stability of the pelvic ring is of great significance for improving joint function, relieving pain, and improving the quality of life after tumor resection. Traditional biologic reconstruction includes iliofemoral fusion, sit-femoral fusion, femoral transposition, and inactivated reimplantation, but these reconstruction modalities are still practiced to have prolonged braking of the lower limbs, non-healing wounds, pseudo joint formation, deep infection and other shortcomings [3–6]; prosthetic reconstruction, on the other hand, includes saddle shaped prosthesis, ice cream cone prosthesis, customized prosthesis, grouped prosthesis, but these prosthetic reconstruction modalities are still faced with limitations such as high tumor resection requirements, difficulty, limited indications, higher incidence of postoperative dislocations, infections, and loosening, and poor matching of prosthesis with patients [7–9].

In recent years, with the development of computer-aided technology and material science, 3D-printing technology provides a new way for accurate matching and osseointegration of pelvic prosthesis, which makes biomechanically-designed prosthesis implantation possible.3D-printed customized prosthesis realizes accurate tumor resection and personalized prosthesis implantation in the resection, repair, and reconstruction of pelvic tumors, which improves the accuracy of the surgical resection of pelvic tumors and at the same time The porous structure of the prosthesis facilitates osseointegration and improves the compatibility between the prosthesis and the bone defect, which results in better long-term stability of the reconstructed pelvic ring [10, 11]. The effectiveness and superiority of 3D-printed customized prostheses in the reconstruction of bone defects after the resection of pelvic tumors has been proven. The present study aims to investigate the role of a 3D-printed titanium alloy integrated prosthesis with a porous interface in the reconstruction of primary and secondary malignant pelvic tumors and in the reconstruction of bone defects in the pelvis. primary and secondary malignant tumors of the pelvis, the main points of application, early efficacy and complications, and to provide a reference for bone tumor specialists in the limb preservation treatment of pelvic malignant tumors.

## Materials and methods

### Study patients

This study strictly complies with data protection regulations, data anonymization measures include: 1) Delete all patients’ names, ID numbers, hospitalization numbers and other direct identifiers; 2) Remove EXIF information and image angle marks in clinical images (X-ray/surgical photos); 3) For minor patients: additional hidden age, date of birth, and guardian information, and replace it with study number; 4) The data set is stored separately from the key, and only the main researchers can access the original mapping table. Between June 2022 and December 2023, we conducted a retrospective analysis of seven patients (4 males, 3 females; mean age 42 years, range 16–54) undergoing 3D-printed titanium custom prosthesis reconstruction for pelvic malignant tumors at our institution. Histologic diagnoses included four primary malignancies (two chondrosarcomas and two osteosarcomas, Enneking stage IIb) and three metastatic carcinomas (primary sites: endometrial, rectal, and hepatocellular). Tumor localization followed the Enneking and Dunham’s type I-IV zoning system [2, 12]: I + II (*n* = 1), I + II + III (*n* = 1), II + III (*n* = 2), I + II + IV (*n* = 2), and I + II + III + IV (*n* = 1). Surgical margins comprised wide resection (*n* = 3), marginal resection (*n* = 3), and intracapsular resection (*n* = 1), with reconstruction methods including standard hemipelvic/iliac prostheses (*n* = 5) and hemi-pelvic prostheses with peg-rod attachment (*n* = 2). Inclusion criteria: (1) Primary malignant bone tumors in the pelvic (osteosarcoma, chondrosarcoma, etc.) or solid tumor oligometastasis (≤ 3 distant metastasis); (2) Primary tumor: Enneking IIA-IIB stage; (3) Metastatic tumor: pelvic metastases with a maximum diameter ≤ 8 cm that do not encase the iliac vessels or sciatic nerve; controlled primary tumor (achieving R0/R1 resection or showing no radiographic progression for ≥ 6 months); and no evidence of pulmonary or other abdominal organ metastases; (4) After tumor resection, bone defects are expected to affect more than 2 pelvic partitions. (5) The operation is completed by the same qualified doctor. Exclusion criteria include: (1) the use of group-coordinated prosthesis or biological reconstruction, including autologous/allogeneic bone graft; (2) the primary tumor has distant metastasis (Enneking III), multiple bone metastasis in metastatic tumors (> 3 places) or combined visceral metastasis; (3) ECOG score ≥ 3 points or expected survival < 6 months (Table 1);

**Table 1:** Demographic characteristics of 7 patients

Case	Age(years)	Gender		Height(cm)	Weight(kg)	Tumour nature	Tumor staging	Tumor zoning	Excision range
1	16		female	165	60	chondrosarcoma	Enneking IIb	I + II	extensive excision
2	33		female	152	52	metastatic adenocarcinoma	TNM IV	I + II + III	marginal excision
3	46		male	173	86	metastatic adenocarcinoma	TNM IV	II + III	marginal excision
4	52		female	160	60	osteosarcoma	Enneking IIb	I + II + IV	extensive excision
5	42		male	170	86	osteosarcoma	Enneking IIb	II + III	extensive excision
6	54		male	178	69	Metastatic liver cancer	TNM IV	I + II + IV	marginal excision
7	53		male	170	70	chondrosarcoma	Enneking IIb	I + II + III + IV	intracapsular resection

### Design and manufacturing of 3D-printed custom prostheses

**Fig. 1:**
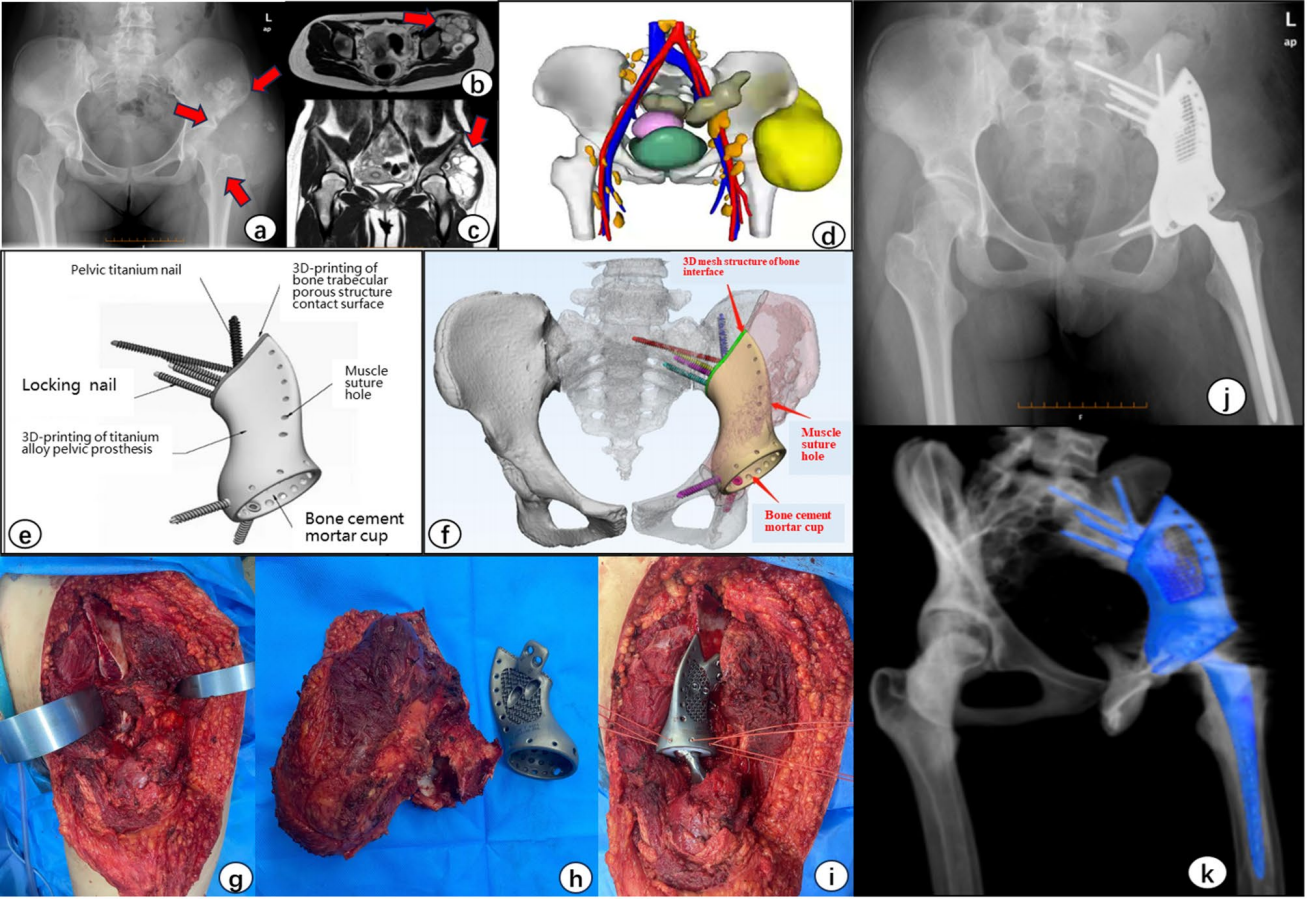
a-c: Preoperative imaging demonstrating tumor invasion into Enneking zones I and II: (**a**) Anteroposterior radiograph; (**b**) Coronal T2-weighted MRI; (**c**) Axial contrast-enhanced MRI. d-f: Surgical planning workflow: (**d**) 3D tumor reconstruction from CT-MRI fusion; (**e**) Prosthesis design schematic highlighting porous interface geometry; (**f**) Virtual osteotomy simulation. g-i: Intraoperative documentation: (**g**) Post-resection cavity; (**h**) 3D-printed titanium prosthesis with anatomic contour matching; (**i**) Prosthesis implantation. **j**-**k**: Postoperative imaging validation: (**j**) Weight-bearing radiograph demonstrating proper screw trajectory; (**k**) CT 3D reconstruction verifying osseous integration at iliac-prosthesis interface and femoral component alignment

**Fig. 2:**
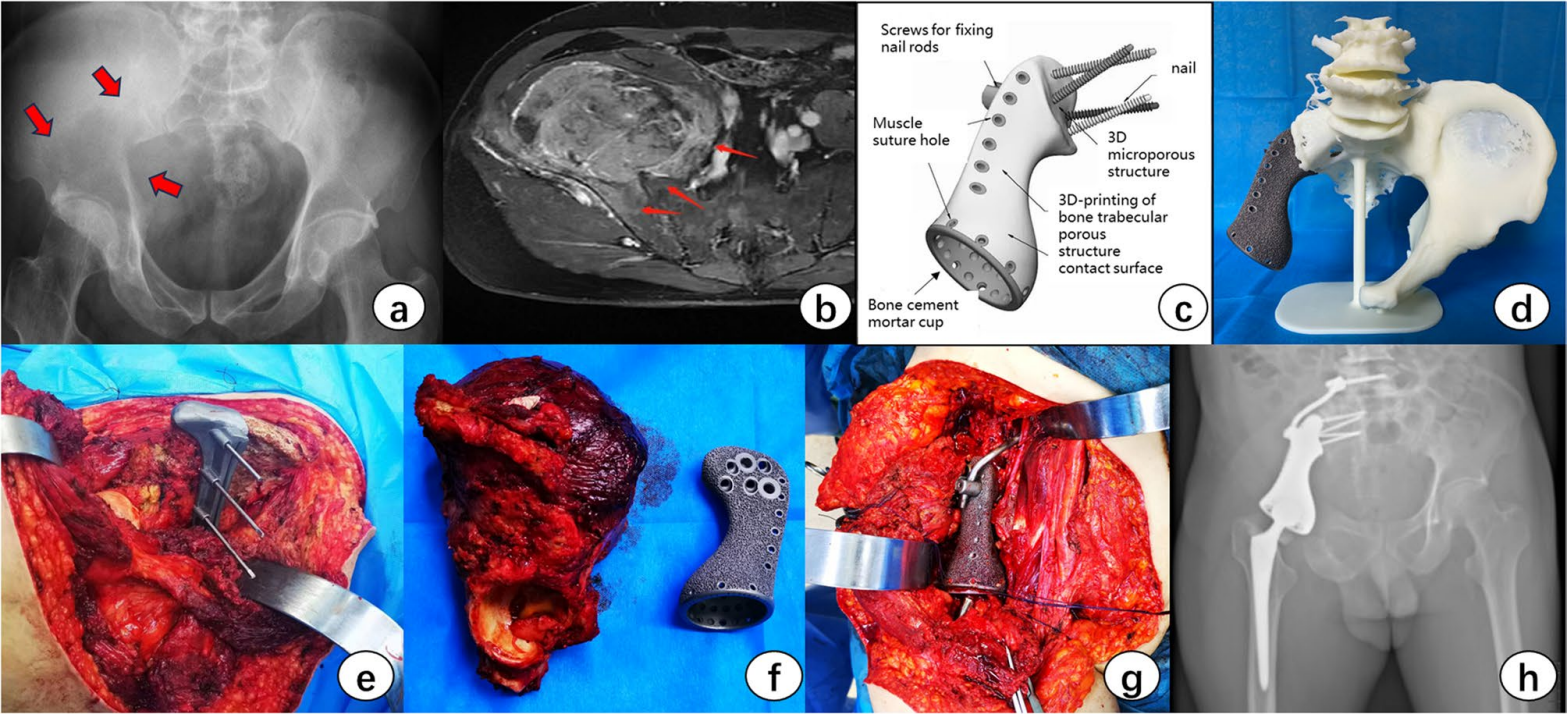
**a**-**b**: Preoperative imaging confirming tumor infiltration in Enneking zones I、II and IV. **c**: Patient-specific 3D-printed titanium prosthesis design based on simulated tumor resection margins. **d**: Preoperative validation using a 1:1 polylactide resection model for prosthesis compatibility testing. **e**: Precision osteotomy guided by a patient-specific osteotomy guideplate. **f**-**g**: Intraoperative verification: (**f**) Comparative assessment of resection cavity and prosthesis geometry; (**g**) Definitive prosthesis implantation with screw-rod fixation system. **h**: Postoperative radiograph confirming optimal prosthesis positioning and sacroprosthetic interface integration

The design of 3D-printed custom prostheses focuses on two critical aspects: determining the resection scope and restoring pelvic stability. Key considerations include patient age, tumor type, growth rate, preservable musculoskeletal soft tissue, sensitivity to adjuvant chemotherapy or radiotherapy, and the mechanical stability of the pelvic ring and joints. Resection planning significantly influences the risk of short-term tumor recurrence. The application of neoadjuvant chemotherapy depends on tumor type. For highly malignant osteosarcoma, neoadjuvant chemotherapy can facilitate R0 resection, reduce intraoperative bleeding, and suppress tumor progression during the prosthesis manufacturing period, thereby avoiding situations where tumor advancement invalidates the intended resection margin. For solitary pelvic metastases, the goal should be marginal excision. Since the prosthesis design and production cycle, including planning, input, output, verification, and confirmation, typically requires 7 to 14 days, resection planning must consider potential tumor progression during this interval. Thus, we recommend adding a 1–2 cm safety margin to the planned resection boundary. The reconstruction of pelvic stability profoundly affects postoperative pain relief, walking posture, joint function, and quality of life. Biomechanical studies indicate that stress concentrations are higher in the sacroiliac joint and the mid-portion of the ilium and its medial edge, whereas the outer pelvic rim and pubic branch experience lower mechanical stress. The design of 3D-printed pelvic prostheses should correspond to these biomechanical characteristics, employing solid and stable fixation in high-stress regions and minimizing material volume in low-stress areas. Guided by these principles, Zone I reconstructions should incorporate a reduced profile in the outer third of the ilium. The medial iliac component should feature a solid high-strength structure, while the lateral aspect may use a porous, trabecular-like lower-strength material. When minimal iliac bone remains, screw fixation should extend into the ipsilateral sacrum (Fig. 1f). If adequate iliac bone is preserved, screws may be placed only within the ilium in transverse and longitudinal directions. Zone II designs should adopt a monolithic solid high-strength structure integrated with a standard acetabular component (Fig. 1e). Reconstruction of Zone III remains debated. Some studies suggest that restoring pelvic ring integrity benefits long-term functional recovery, but reconstruction in this zone carries a substantial risk of mechanical complications such as breakage and loosening. One case in this series experienced pubic screw fracture, likely due to fatigue failure under prolonged gait loading; therefore, we generally advise against reconstruction of the pubic or ischial branches.For lesions involving Zone IV, fixation should be tailored to the degree of bilateral sacroiliac joint involvement, often supplemented with lumbar spinal screw fixation. The iliac wing section of the prosthesis should include screw holes for rod linkage to lumbar screws (Fig. 2c). Additional screw positions should be adapted to the residual bone structure, aiming for interlocking fixation within the ilium where feasible. Screws should traverse the sacroiliac joint when necessary. A trabecular-like porous structure at the bone-prosthesis interface of the sacroiliac joint can promote long-term stability of the pelvic ring (Fig. 2h). Additionally, a polylactide model of the pelvis should be printed to assess the accuracy of the planned osteotomy and the fit of the final titanium prosthesis preoperatively (Fig. 2d and 2f).

The custom 3D-printed prostheses used in this study were designed by our team. Patient CT data, stored in DICOM format, were imported into Materialise Mimics V25.0 software (Materialise Corp, Belgium) to generate virtual 3D reconstructions of the tumor and pelvic anatomy. Adequate surgical margins were determined digitally, and patient specific osteotomy guides were designed according to the intended cutting planes to facilitate accurate bone resection during surgery. Prosthesis and guide design were performed using 3-matic V17.0 software (Materialise Corp, Belgium). The implant was engineered to precisely fit the bone defect, with extraneous features removed using Geomagic Wrap 2017 software (Geomagic Inc, Morrisville, USA). The bone implant interface was designed as a trabecular like 3D-printed mesh structure to promote long term osseointegration, while the prosthesis and its flange plate incorporated multiple screw channels for primary stability. The hemipelvic prostheses were fabricated from medical grade Ti6Al4V powder using an electron beam melting system (Arcam EBM Q10plus, GE Additive, Sweden). Surgical guides were produced from medical grade PA12 powder using a non metal 3D printing system (EOS P110, EOS GmbH, Munich, Germany) (Figs. 1e and 1f and 2c and 2e).

### Surgical method

Following the induction of general anesthesia, the surgical incision and patient positioning were determined based on tumor location and size. All 7 patients in this study underwent a combined anterior and posterior surgical approach, with the procedures performed by the same qualified surgeon. Critical steps included tumor resection according to the preoperative plan, accurate placement of the osteotomy guides, and stable fixation of the 3D-printed prosthesis. Preoperative embolization of the internal iliac artery was essential to minimize bleeding. For highly vascularized tumors, temporary loop ligation of the abdominal aorta may be considered intraoperatively if necessary. During resection involving the Zone III pubic or ischial bones, a sufficiently extended curvilinear incision along the lateral border of the gluteus maximus was crucial to adequately expose the ischial tuberosity due to its deep anatomical location. Inadequate exposure could prolong operative time, increase intraoperative bleeding, and elevate the risk of surgical site infection. During dissection and osteotomy of the pubic branch, protection of the external iliac vessels, femoral nerve, and obturator neurovascular bundle was imperative, particularly in patients of reproductive age. A recommended technique involves dissecting along the superior pubic ramus toward the pecten pubis after exposing the internal iliac plate, carefully separating the iliopsoas muscle bundle enveloping the neurovascular structures. Unless infiltrated by tumor, isolated dissection of the neurovascular bundle within the iliopsoas is not advised, as it may increase iatrogenic injury and remove a protective barrier in case of local recurrence.For Zone IV resections, protection of the lumbosacral plexus was paramount. Magnetic resonance neurography (MRN) provided superior visualization for assessing the proximity between the tumor and the lumbosacral neural structures (Fig. 2b).

Precision osteotomy is critical for the successful implantation of 3D-printed custom prostheses. As the prosthesis contact surface, screw dimensions, and acetabular orientation are all determined during the planning phase, executing the osteotomy exactly as planned is essential for achieving accurate prosthesis placement. Thorough soft tissue mobilization around the target area is instrumental in ensuring the correct positioning of the osteotomy guide (Fig. 2e). Following resection, the excised bone specimen was compared with the 3D-printed model to confirm the precision of the osteotomy (Figs. 1h and 2f). In cases where more bone than anticipated was resected, the autologous femoral head can be utilized as a graft to promote integration at the bone-prosthesis interface. Given that pubic screw fixation is uniaxial, our protocol recommends first securing the prosthesis with multi-directional iliac or sacral screws—employing a combination of transverse and longitudinal trajectories—to establish primary stability. This approach minimizes the risk of inadequate fixation resulting from subsequent adjustments. For Zone IV resections, supplemental screw placement in the L5 and/or L4 vertebrae is necessary. Screw fixation at these levels can be achieved using either a pedicle-based technique or a direct lateral approach into the vertebral body, 3 relevant cases in this study utilized lateral vertebral body screws, which were connected to the prosthesis using metal rods (Fig. 2h).The integrated design of the prosthesis obviates the need for intraoperative modifications to acetabular position, as the patient-specific bone-implant interface is engineered to inherently provide optimal component alignment. A polyethylene oversized liner was subsequently cemented into the metal acetabular shell, followed by implantation of the femoral component and reduction of the hip joint (Fig. 1j).Muscular reconstruction is crucial for functional recovery. The iliac wing segment of the prosthesis should be designed with suture holes to facilitate soft tissue reattachment. Although tumor resection frequently entails significant loss of musculoskeletal tissue, every effort should be made to reconstruct the gluteus medius and minimus muscles onto the prosthesis to prevent Trendelenburg gait (Fig. 1i). Prior to wound closure, extensive irrigation with povidone-iodine saline solution is imperative, as this practice has proven more effective in reducing the risk of deep surgical site infection than postoperative administration of broad-spectrum antibiotics alone.

### Rehabilitation exercise

Initiating functional exercise with appropriate intensity at the correct time is essential not only for maximizing hip functional recovery and facilitating a return to normal daily activities, but also for preventing complications such as prosthesis dislocation and thrombosis. Generally, due to the loss of musculoskeletal tissue, the hip joint remains unstable during the first 2–3 weeks postoperatively. During this period, patients are advised to wear a pelvic–lower limb brace fixed at 15°–25° of hip abduction, 15° of hip flexion, and 15° of knee flexion. Isometric muscle contractions should be performed in bed to enhance hip muscle strength and balance. Patients may begin standing without weight-bearing at 3 weeks post-surgery, progressively increase weight-bearing hip flexion exercises at 4 weeks, and advance to walking with a assistive device between 6 and 8 weeks. In cases with extensive muscular deficit, the entire rehabilitation protocol should be adjusted accordingly.

### Evaluation index

The VAS and KPS score were used to evaluate the pain level and physical strength difference of the patients preoperatively(B.S), at 1 month(1 month A.S), 3 months (3 months A.S) and 6 months (6 months A.S) after surgery, and the MSTS 93 score was used to evaluate the limb function of the patients before surgery (MSTS 93 B.S)and at 3 months after surgery (MSTS 93 A.S). The patients were grouped according to whether the tumor involved zone IV or not (Lesions involving zone IV of the Enneking and Dunham classification were classified as the “IV group”, and all others as the “Non-IV group.”), and the healthy sacroiliac joint gap at the level of the first sacral neural foramen was measured under CT to evaluate sacroiliac joint stability preoperatively and 6 months postoperatively, respectively.

### Follow-up

During the first two years postoperatively, patients underwent CT scans of the chest and pelvis every three months and radionuclide imaging every six months. Monthly telephone follow-up was conducted to monitor survival status. Overall survival (OS) was calculated from the date of surgery until death from any cause. Progression-free survival (PFS) was defined as the time from surgery to the first occurrence of either local recurrence or distant metastasis. All postoperative complications were systematically recorded, including prosthesis dislocation, periprosthetic osteolysis, heterotopic ossification, screw loosening, screw fracture, and infection.

### Statistical methods

Statistical analysis was performed using SPSS software (version 25.0; IBM Corp., Armonk, N.Y., USA). Since the VAS and KPS scores did not follow a normal distribution as assessed by the Shapiro–Wilk test, they are described using medians and interquartile ranges (IQR). The Friedman test was employed for comparisons across multiple groups. If the Friedman test indicated a significant overall difference, Dunn’s post hoc test was applied for pairwise comparisons, with P-values adjusted using the Bonferroni correction to control for multiple testing. The significance level was set at α = 0.05.After normality was evaluated with the Shapiro–Wilk test, the MSTS 93 score (W = 0.913, *P* = 0.420) and sacroiliac joint space (W = 0.886, *P* = 0.256) were found to be normally distributed; therefore, paired t-tests were used for comparisons, with results expressed as mean differences along with their 95% confidence intervals. Survival curves were plotted using the Kaplan–Meier method, and a multivariate Cox proportional hazards regression model was applied to analyze factors influencing progression-free survival.

## Results

All patients were completed according to the preoperative plan, satisfactory margins were obtained in 6 patients (85.7%), 1 patient was preoperatively planned for intracapsular resection due to tumor involvement of the whole pelvis and femur, thus his resection did not involve the sacroiliac joints, 1 patient with metastatic carcinoma was found to have supragluteal artery aneurysm embolism, and 2 patients with sacroiliac joints invasion underwent reconstruction with screw-connected hemi-pelvic prostheses, 7 patients Surgical time was 6–10 h (mean 7.8 h), intraoperative bleeding was 870–13300 ml (mean 3981.42 ml), and follow-up was 6–18.2.2 months (mean 10.6 months). Postoperative CT review showed complete integration of the prosthesis with the preserved bone.

### VAS and KPS score

**Fig. 3:**
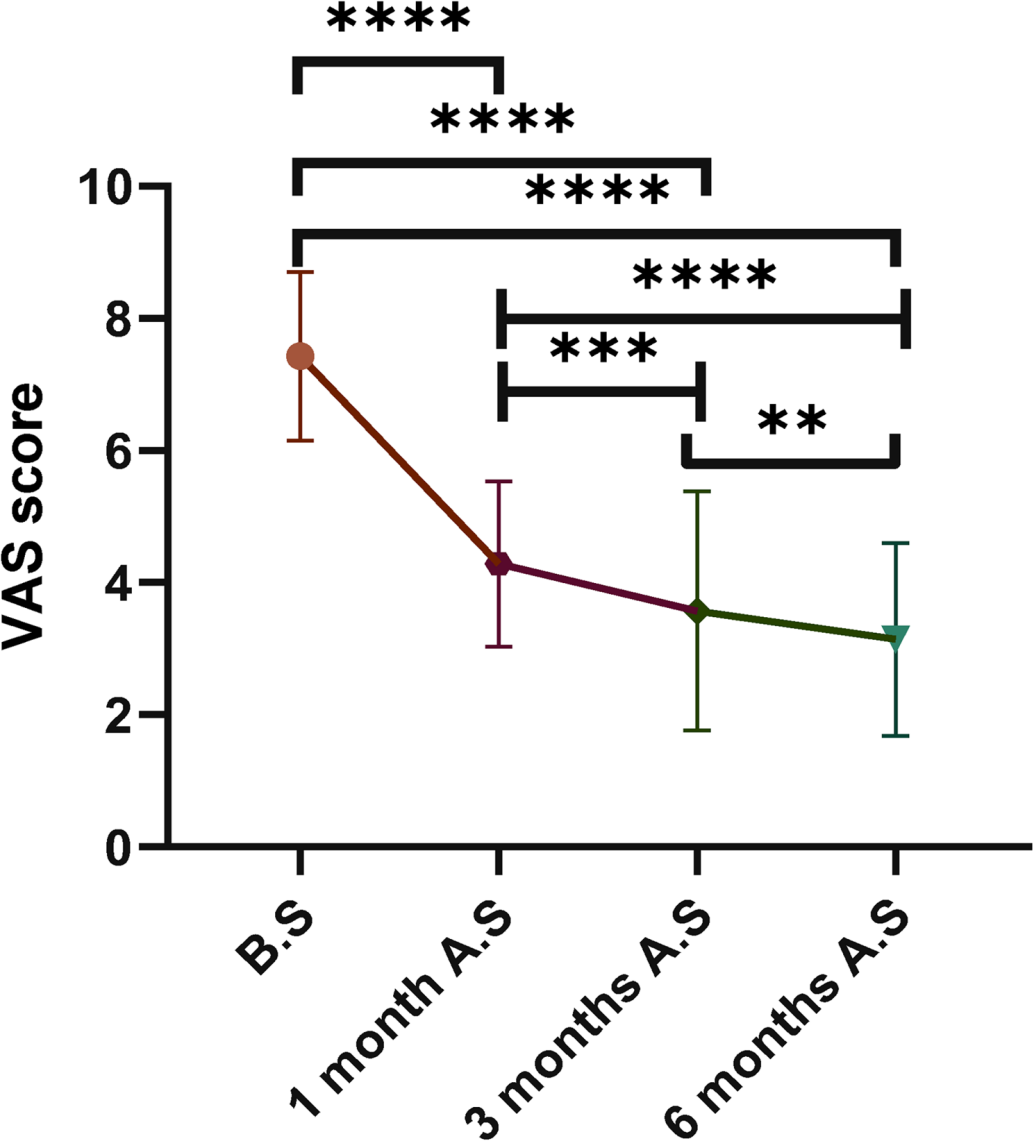
Comparison of VAS scores at different time

VAS: The postoperative pain VAS scores demonstrated a consistent downward trend. The median preoperative score was 7.0 (IQR: 6.0–8.5), which decreased to 4.0 (3.5–5.0) at 1 month, further declined to 3.0 (2.0–4.5) at 3 months, and reached its lowest point of 2.5 (2.0–3.5) by 6 months postoperatively. The Friedman test revealed a statistically significant difference in VAS scores across the four time points (χ² = 9.46, *P* = 0.024). Post hoc pairwise comparisons with Bonferroni correction indicated that the preoperative pain score was significantly higher than those at 1 month (*P* < 0.001), 3 months (*P* < 0.001), and 6 months (*P* < 0.001). Additionally, the score at 1 month was significantly higher than those at 3 months (*P* = 0.002) and 6 months (*P* < 0.001). A statistically significant difference was also observed between the scores at 3 months and 6 months postoperatively (*P* = 0.002) (Fig. 3; Table 2).

**Fig. 4:**
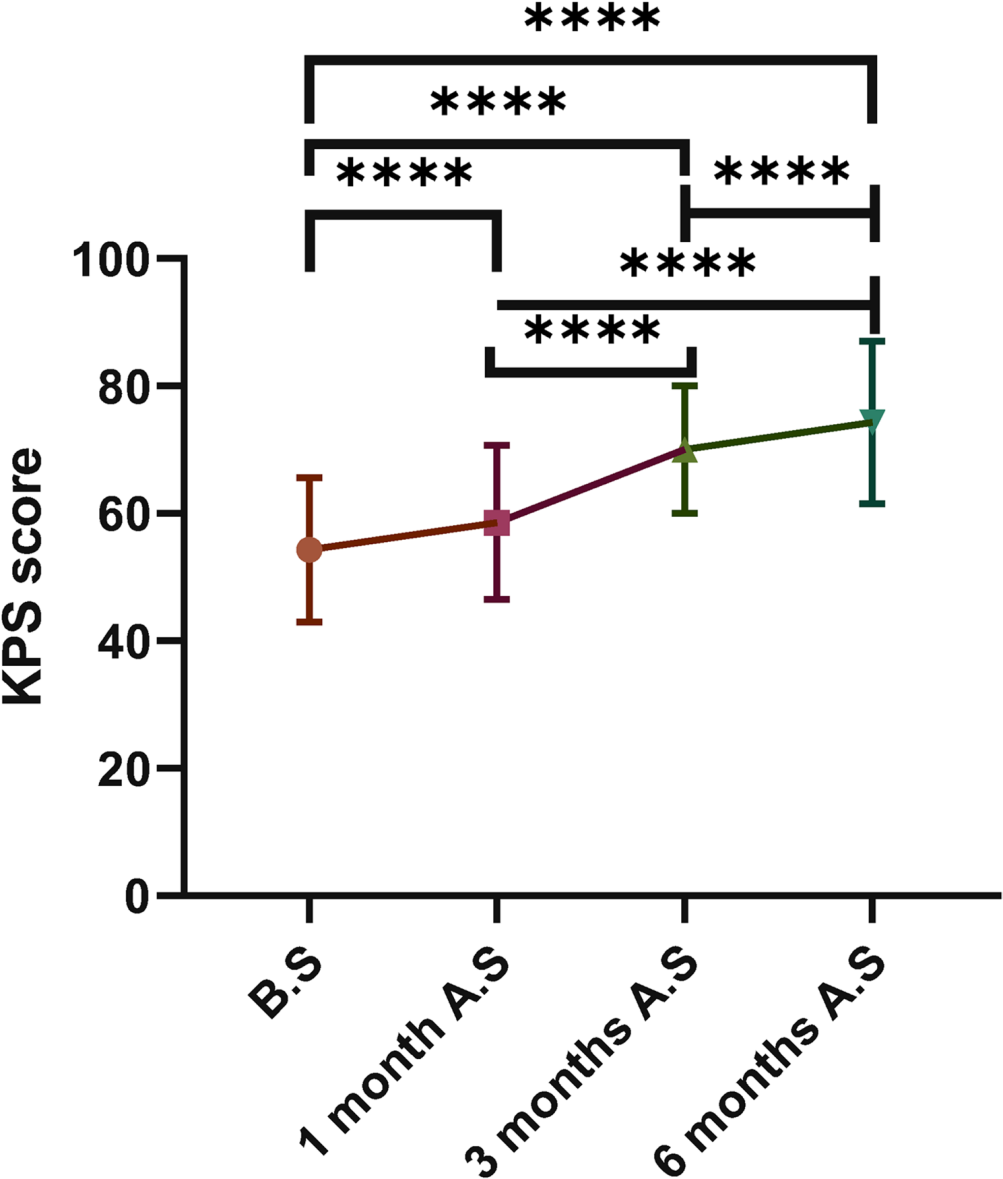
Comparison of KPS scores at different time

KPS: A significant difference was observed in KPS scores between preoperative and postoperative assessments (χ² = 13.36, *P* = 0.004). Dunn’s post hoc test with Bonferroni correction revealed that all pairwise comparisons between time points were statistically significant (*P* < 0.001). The median KPS score showed continuous improvement from 60.0 (IQR: 40.0–60.0) preoperatively to 60.0 (50.0–70.0) at 1 month, 70.0 (60.0–80.0) at 3 months, and 80.0 (70.0–80.0) at 6 months postoperatively (Fig. 4; Table 2).

**Table 2:** Comparison of VAS and KPS scores at different time points

Score	Comparison	Z value	*P*	95%CI	Significance
VAS	B.S vs. 1 month A.S	16.67	< 0.001	(−4.0, −2.0)	****
	B.S vs. 3 months A.S	21.74	< 0.001	(−5.0, −3.0)	****
	B.S vs. 6 months A.S	25.36	< 0.001	(−6.0, −4.0)	****
	1 month A.S vs. 3 months A.S	5.07	< 0.001	(−2.0, 0.0)	***
	1 month A.S vs. 6 months A.S	8.69	< 0.001	(−3.0, −1.0)	****
	3 month A.S vs. 6 months A.S	3.62	0.0018	(−1.0, 0.0)	**
KPS	B.S vs. 1 month A.S	−7.97	< 0.001	(0.0, 20.0)	****
	B.S vs. 3 months A.S	−20.29	< 0.001	(10.0, 30.0)	****
	B.S vs. 6 months A.S	−25.36	< 0.001	(20.0, 30.0)	****
	1 month A.S vs. 3 months A.S	−12.32	< 0.001	(0.0, 20.0)	****
	1 month A.S vs. 6 months A.S	−17.39	< 0.001	(10.0, 20.0)	****
	3 month A.S vs. 6 months A.S	−5.07	< 0.001	(0.0, 10.0)	****

Significance levels in the figures or tables are denoted as follows: **** for *P* < 0.0001, *** for *P* < 0.001, and ** for *P* < 0.01

### MSTS 93 score

**Fig. 5:**
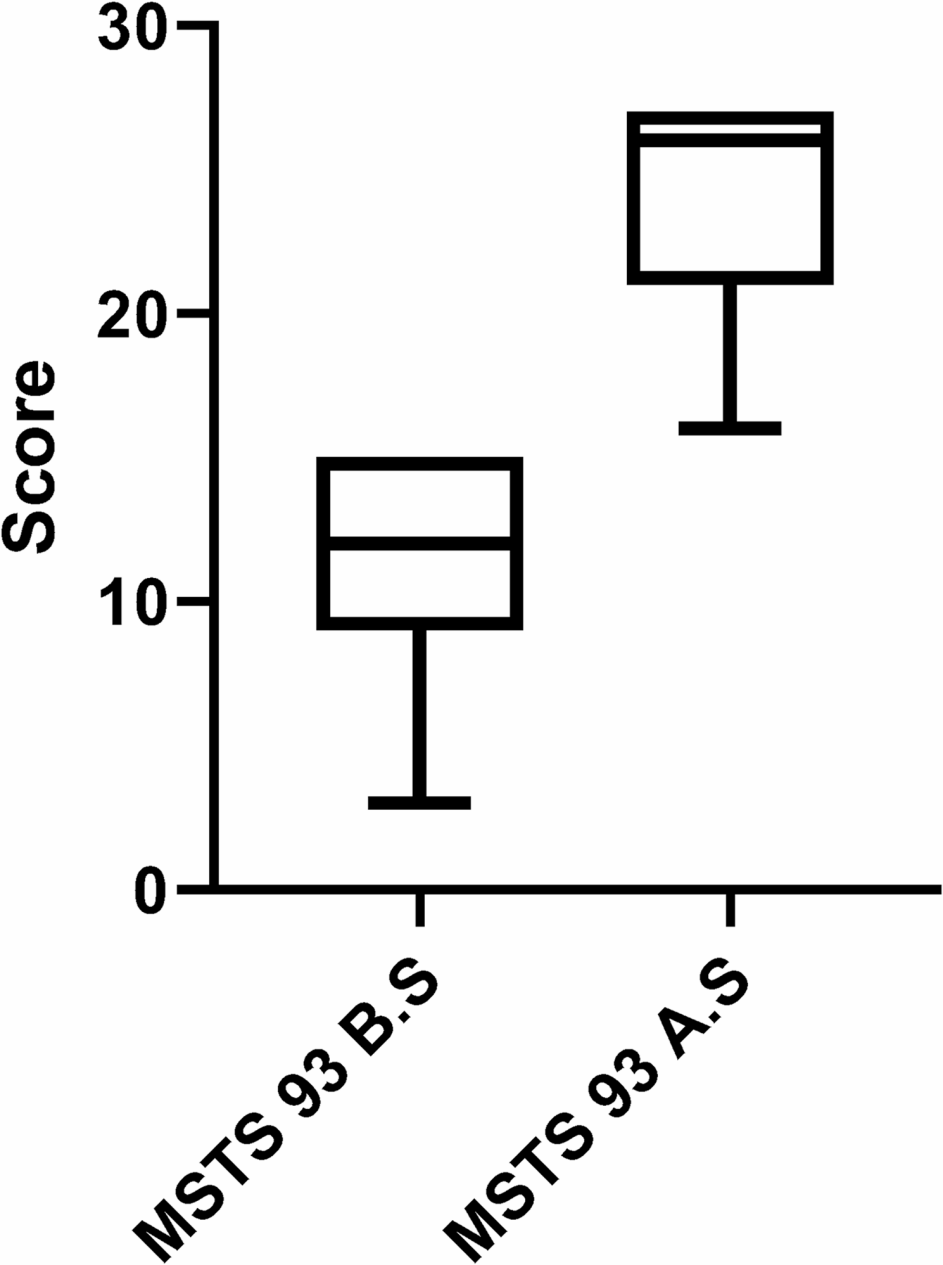
Distribution of MSTS 93 score before and after surgery

**Fig. 6:**
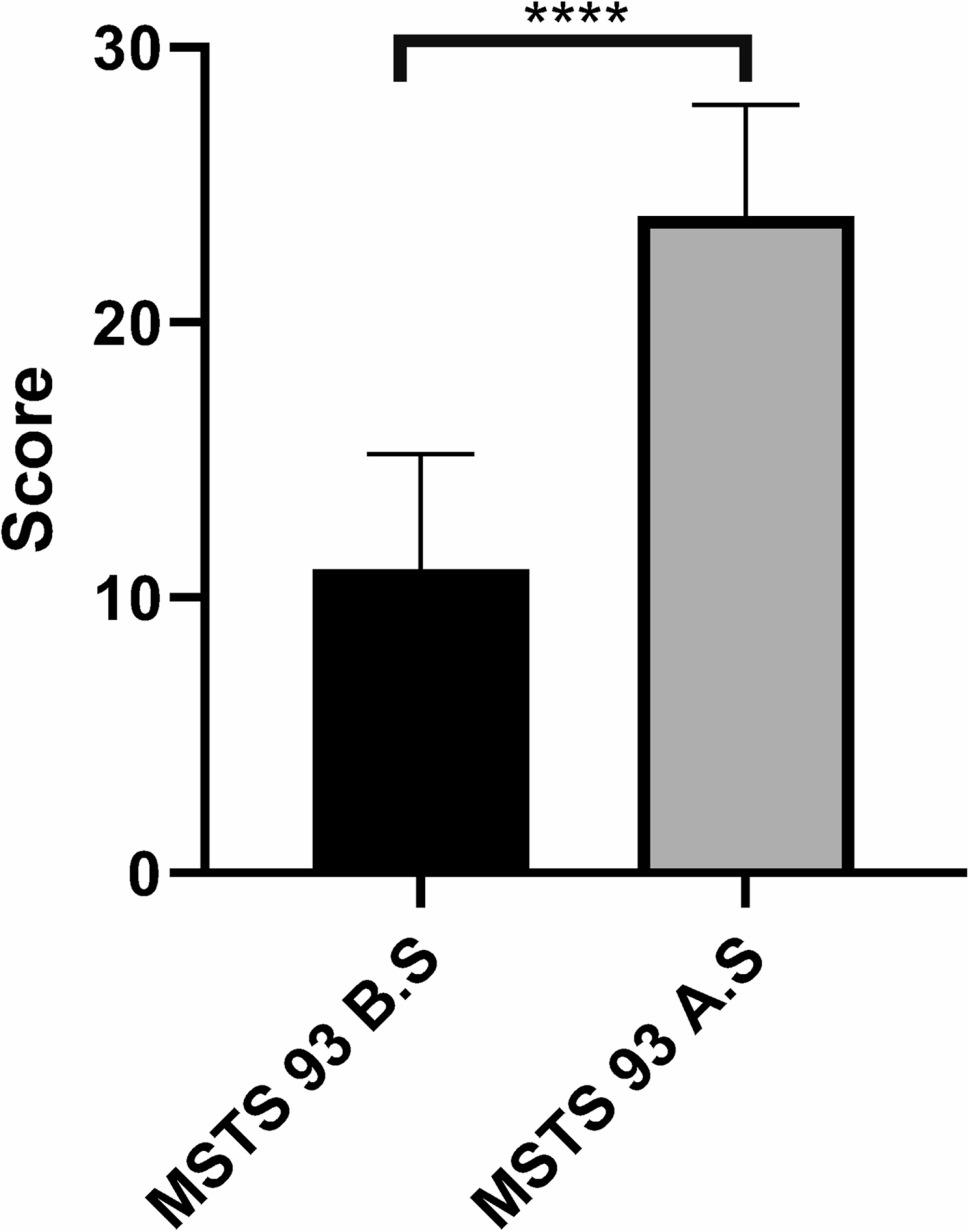
Mean value and comparison of MSTS 93 score before and after surgery

All 7 patients were evaluated for MSTS 93 scores preoperatively and 3 months postoperatively (Fig. 5), with MSTS 93 scores of 151.00 ± 4.20 preoperatively and 23.86 ± 4.06 at 3 months postoperatively, with a statistically significant difference between the two (*P* < 0.001, 95% CI: 11.40,14.31) (Fig. 6).

### Stability of sacroiliac joint

**Fig. 7:**
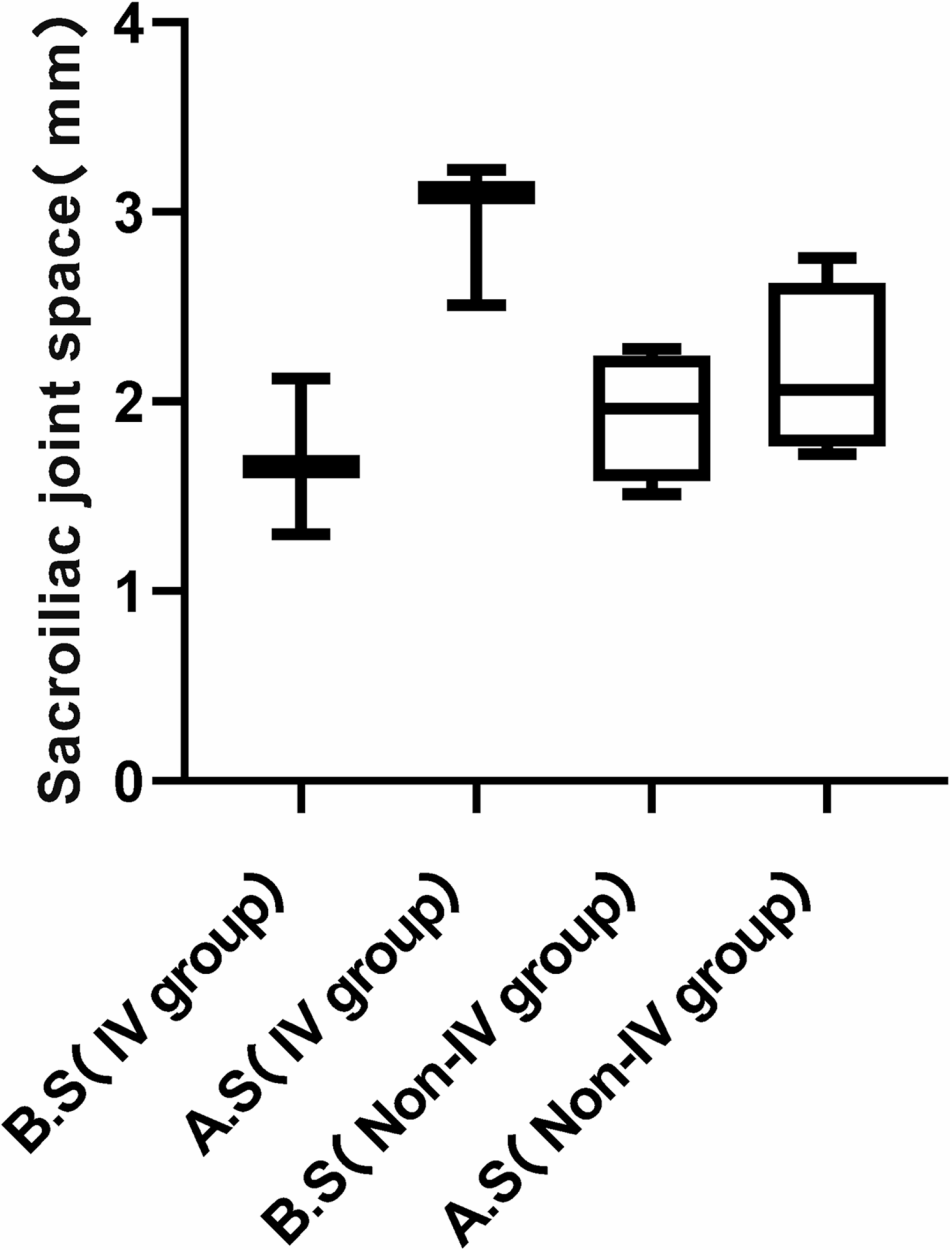
IV group and Non-IV group in sacroiliac joint space before and after surgery

**Fig. 8:**
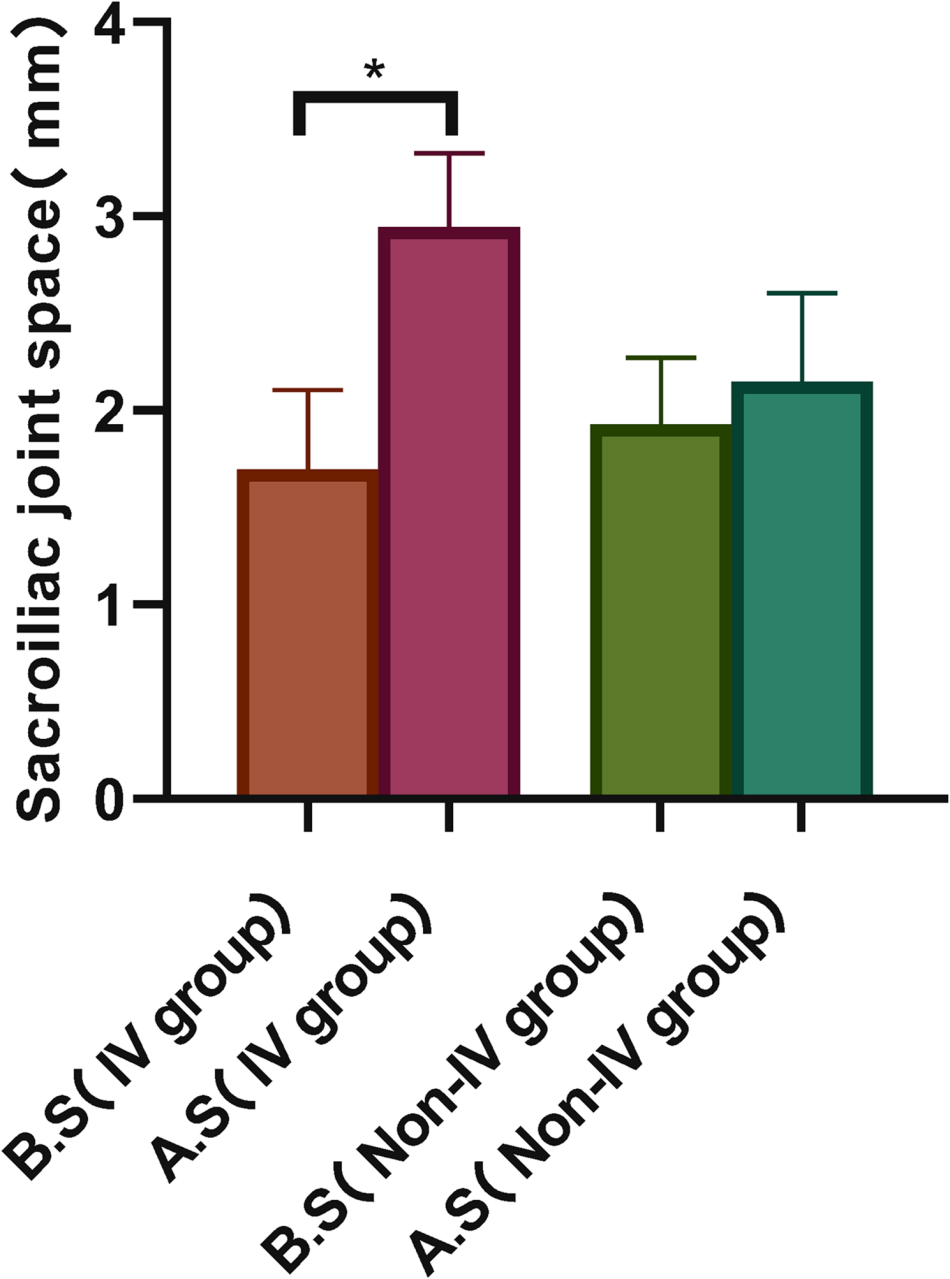
Comparison of sacroiliac joint space before and after operation

Patients were classified into an IV group and a Non-IV group based on whether the lesion involved Zone IV. The contralateral sacroiliac joint space was measured preoperatively and postoperatively in both groups (Fig. 7). In the IV group, the joint space measured 1.69 ± 0.41 mm preoperatively and 2.94 ± 0.38 mm postoperatively, demonstrating a statistically significant difference (*P* = 0.048; 95% CI: −2.47 to −0.03). In contrast, the Non-IV group showed measurements of 1.92 ± 0.34 mm preoperatively and 2.15 ± 0.45 mm postoperatively, with no statistically significant difference observed (*P* = 0.089; 95% CI: −0.51 to 0.06) (Fig. 8).

### Survival analysis

**Fig. 9:**
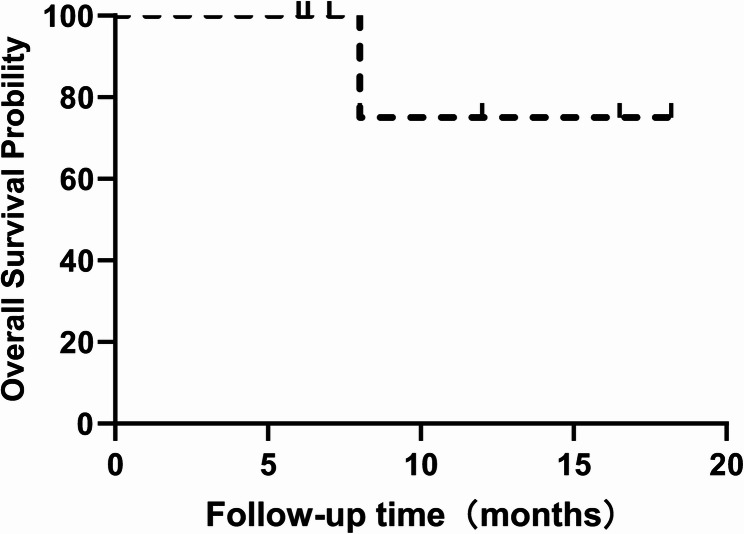
Overall survival curve

**Fig. 10:**
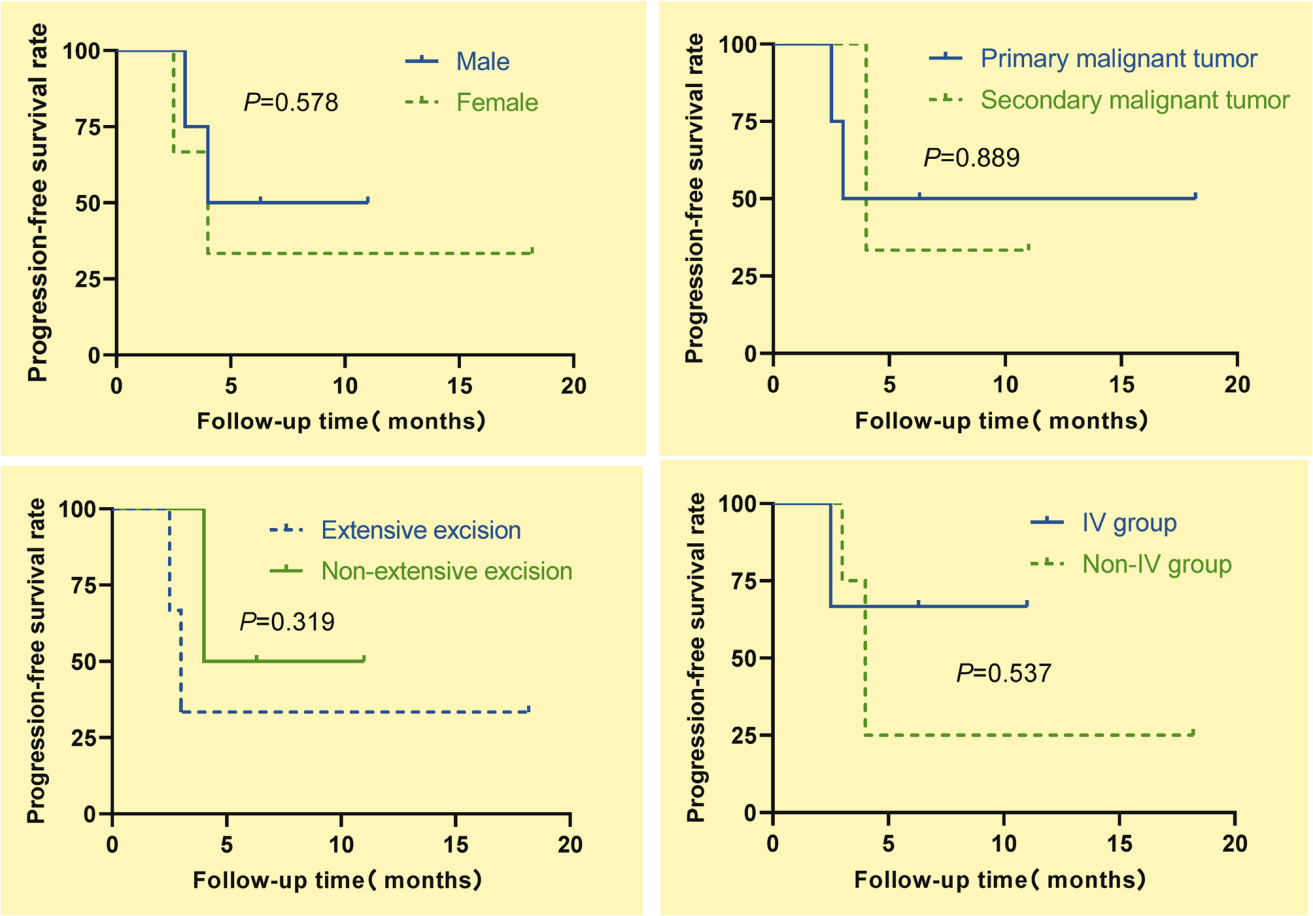
Progression-free survival analysis curve

During the follow-up period, one of the seven patients died from tumor recurrence and metastasis (Fig. 9). Progression-free survival (PFS) was analyzed by tumor type, gender, resection margin status, and tumor location. The median PFS was 4.0 months for both males and females, with no statistically significant difference between genders (*P* = 0.578). Patients with primary malignant tumors had a median PFS of 3.0 months, compared to 4.0 months in those with metastatic cancer (*P* = 0.889). The R0 resection group had a median PFS of 3.0 months, while the R1 resection group showed a median PFS of 4.0 months, though this difference was not statistically significant (*P* = 0.319). The median PFS was not reached in the IV group, whereas the Non-IV group had a median PFS of 4 months (*P* = 0.537) (Fig. 10). A Cox proportional hazards regression model with Firth correction including all these variables identified no significant factors associated with PFS(*P* >0.05) (Table 3).

**Table 3:** COX multivariate regression analysis of the progression of 7 patients

	B	SE	Sig	Exp(B)	95%CI for Exp(B)
Gender	−0.147	1.224	0.886	0.863	0.103–7.468
Excision range	−1.565	2.369	0.384	0.209	0.001–6.585
Tumor type	−0.903	2.080	0.561	0.405	0.003–7.964
Zoning	−0.205	1.294	0.847	0.814	0.108–9.282

### Complication

Complications among the seven patients included heterotopic ossification in four cases (57.1%), fracture of the pubic screw in one (14.3%), which remained asymptomatic and did not affect ambulation, and femoral neck dislocation in one patient (14.3%) resulting from improper rehabilitation exercises. One patient (14.3%) experienced delayed wound healing, two (28.6%) developed local recurrence, new metastatic lesions developed in two patients (28.6%). Critically, no deep infections, periprosthetic osteolysis, or implant loosening were observed in any patient (Table 4).

**Table 4:** Complications and treatment of all patients

Patient number	Complication	Time of occurrence(month)	Treatment methods
1	Heterotopic ossification	4	None
1	Fracture of pubic nail	15	None
2	Local recurrence	4	Radiotherapy
3	Dislocation of neck of femur	1	Secondary surgical reduction
4	Heterotopic ossification	3	None
4	Delayed healing	1	Debridement operation、
4	metastases	2	chemotherapy
5	Heterotopic ossification	3	None
5	Local recurrence	3	Radiotherapy and
5	metastases	3	chemotherapy
6	Heterotopic ossification	4	None

## Discussion

The complex anatomy of the pelvis and challenges in bone defect reconstruction have historically presented significant difficulties in limb-preservation surgery for malignant pelvic tumors. Recent advancements in computer simulation software and materials science have established 3D-printed custom prostheses as a valuable approach for reconstructing pelvic defects following tumor resection. This study describes the technical points and early clinical outcomes of 3D-printed titanium hemipelvic prosthesis replacement, focusing on three critical aspects: prosthetic design, tumor resection with reconstruction, and postoperative rehabilitation. The results demonstrated significant pain relief and improved quality of life at various postoperative time points. The mean MSTS 93 score reached 23.86 ± 4.06 at three months post-surgery. Reported complications highlight important limitations of the technique; notably, one case of pubic screw fracture has led to a more cautious approach regarding Zone III reconstruction. Furthermore, following prosthetic fixation of the involved sacroiliac joint, the contralateral sacroiliac joint space showed measurable enlargement compared to preoperative status. Survival analysis using a Cox proportional hazards model indicated that gender, tumor type, resection margin, and sacroiliac joint invasion did not significantly affect progression-free survival.

**Fig. 11:**
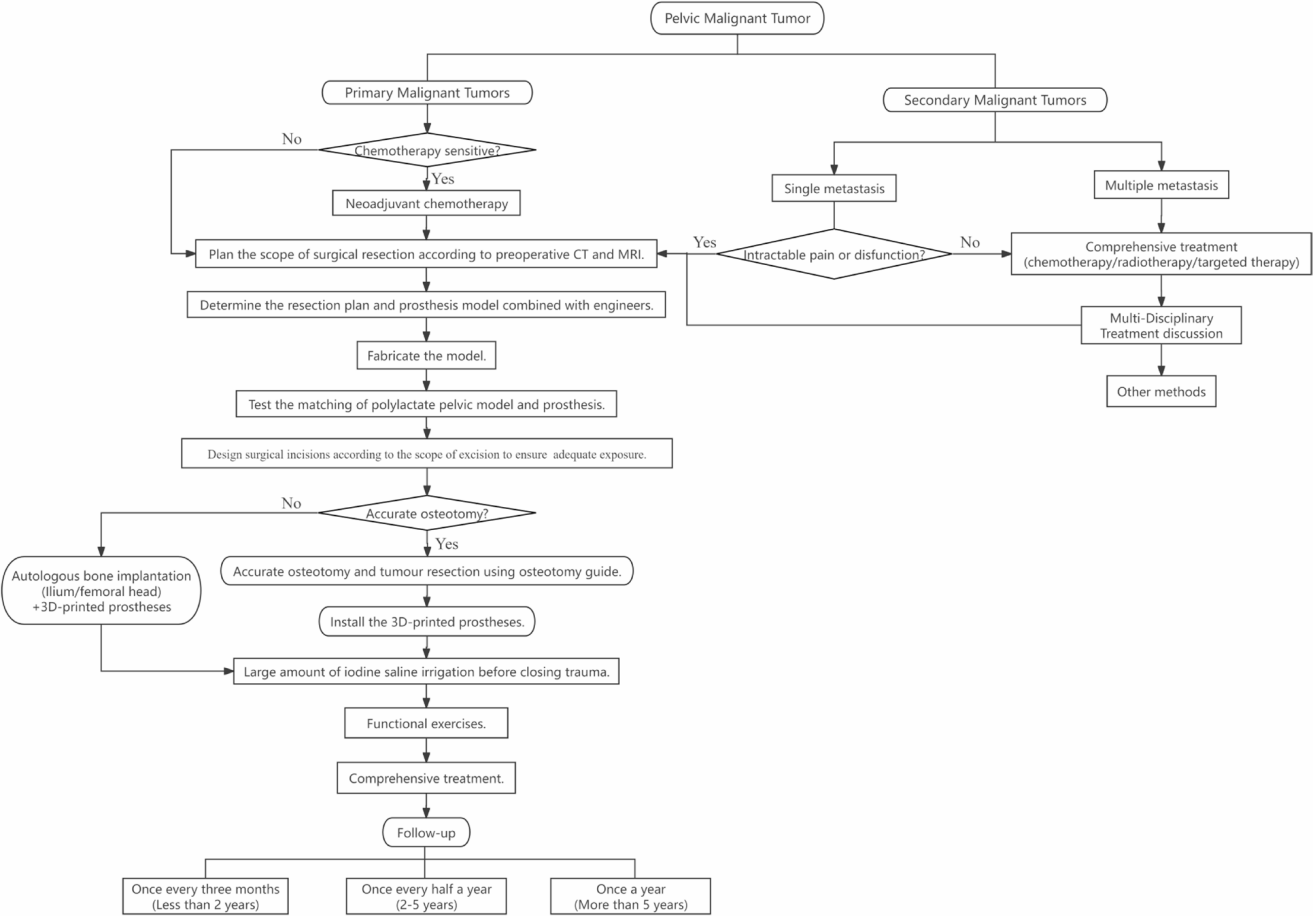
Treatment process of pelvic malignant tumors

Saddle prostheses demand substantial residual iliac bone and exhibit high complication rates. Subsequent designs like custom implants and the Pedestal Cup reduced mechanical failure but were plagued by significant infection risks. Although modular systems offered better fit and functional outcomes, achieving accurate intraoperative adaptation remained a challenge [24–26]. The advent of 3D-printed introduced a novel approach to enhancing integration between the implant and host bone. The 3D-printed porous metallic trabecular structure is now regarded as the gold standard for bone-implant interface biological integration [10, 27]. Subsequently, 3D-printed custom hemipelvic prostheses have been implemented widely. In addition to their superior osteointegration properties, this reconstruction approach resolves the historical conflict between maximizing native bone preservation and achieving complete tumor resection based on 2D imaging. Compared to modular 3D-printed prostheses, monolithic custom designs feature preoperatively planned screw holes that simplify intraoperative installation and fixation. The creation of anatomically accurate 3D-printed tumor models enables more detailed surgical planning [28], and the development of the digital chain of osteotomy guides production makes it easier to accurately resect the pelvic tumors [29]. These techniques provide surgeons with the ability to reduce surgical time and bleeding. The decision to proceed with 3D-printed pelvic prosthesis replacement is contingent upon a comprehensive evaluation of each patient’s eligibility. As outlined in our treatment pathway (Fig. 11), this assessment is critical for maximizing the success of limb salvage surgery. For cases where patients are deemed unsuitable, the algorithm also directs clinicians toward appropriate alternative personalized treatment options. In related applied research, Wu et al. [23] documented early functional outcomes in 28 patients undergoing reconstruction with 3D-printed personalized prostheses, reporting an MSTS 93 score of 23.2, a complication rate of 32%, and satisfactory osseointegration at the bone–prosthesis interface. Our results are consistent with these findings, with an MSTS 93 score of 23.86 ± 4.06 and a prosthesis-related complication rate of 28.6%. Furthermore, although non-parametric tests were used for analyzing VAS and KPS scores in our cohort, the consistent improvement trends observed are in line with those described in other studies utilizing 3D-printed custom prostheses for malignant pelvic tumors [6, 20, 21]. However, the local recurrence rate (42.8%) was higher than that reported in other studies, which may be attributed to the inclusion of patients with metastatic pelvic cancer and the limited sample size. In contrast to previous prosthetic reconstruction methods, no deep infections were observed in this series. In addition to the implant material properties, both meticulous irrigation of the surgical site and adequate soft tissue coverage may have played a critical role in preventing this complication. Feng et al. [13] reconstructed the pelvis using 3D-printed custom hemipelvic prostheses in 10 patients with malignant pelvic tumors. Postoperative VAS and MSTS 93 scores were 2.2 ± 0.9 and 19.4 ± 5.9, respectively, both improved compared to preoperative values. The dislocation rate was 20%, delayed wound healing occurred in 30%, and the infection rate was 20%. The study suggested that dislocation and wound-related complications were associated with tumor involvement of the iliopsoas and gluteus medius muscles, which is consistent with our view on the importance of gluteus medius reconstruction. Liang et al. [10] reported 35 patients who underwent pelvic reconstruction with 3D-printed prostheses. Mean MSTS 93 scores were 22.7, 19.8, and 17.7 for patients receiving iliac, standard, and screw-connected prostheses, respectively. The delayed wound healing rate was 20%, slightly higher than our result (14.7%), while the dislocation rate of 5.7% was lower than our observed 14.7%. These discrepancies may be attributable to the relatively small sample size in our study. Broekhuis et al. [13] documented outcomes from 15 patients receiving 3D-printed custom titanium pelvic prostheses. Their median MSTS 93 score was 63.3%, with an implant-related complication rate of 26.6%. Rates of local recurrence, hip dislocation, and deep infection were each 6.7%, and structural complications reached 20%. While these complication rates appear higher than those in our study, it is noteworthy that their mean follow-up was considerably longer, at 33.8 months, and two benign tumors were included in their cohort. These factors likely contribute to the observed differences in outcomes. Studies on complications following Zone III reconstruction remain limited, and whether reconstruction should be performed is still debated. In this study, one case of screw fracture at the pubic branch was identified during imaging review at 15 months postoperatively. Although this complication did not cause pain or gait impairment in the patient, it nonetheless suggests that screw fixation alone may not ensure long-term stability of the anterior pelvic ring. Paredes et al. [27] reported a case of isolated reconstruction with a 3D-printed prosthesis after pubic osteotomy, which showed good results but did not record any complications related to long-term follow-up, it can be seen that more research on area III reconstruction needs to be further explored. Maintaining sacroiliac joint stability is critical following Zone IV tumor resection. In our reconstructions involving this zone, we adopted the technique proposed by Zhang et al. [30], using a trans-lumbar fixation method with a custom hemipelvic prosthesis. In addition to placing 4–5 screws laterally into S1 and S2, the prosthesis was connected to the lumbar spine with rod fixation based on the “triangular stability principle.” Postoperative evaluation revealed that while patients with tumor involvement of the sacroiliac joint achieved stability on the affected side through screw fixation and osseointegration of the 3D-printed implant, subtle separation tendencies were observed over time in the contralateral unaffected sacroiliac joint under prolonged gait loading. This suggests a hypothesized risk of instability in the healthy-side joint under long-term weight-bearing conditions following prosthetic stabilization. Lv et al. [16] performed reconstruction using 3D-printed customized prostheses in 6 patients with tumors involving the sacroiliac joint (Enneking I + IV). Unlike our approach, their technique did not involve rod connection between lumbar screws and the prosthesis, but rather relied solely on multiple cancellous screws inserted into the sacrum (S1 and S2) for fixation, depending entirely on full osseointegration between the implant and the ilium and sacrum for stability. Their reported mean MSTS score was 25.33 and mean VAS score was 1.33, indicating functional and pain improvement outcomes comparable to ours. However, that study did not include further evaluation of sacroiliac joint stability. Although Liang et al. [10] reported bilateral sacroiliac joint stability following the application of 3D-printed hemipelvic prostheses in a comparative study, their cohort consisted of patients with Zone II or Zone II + III involvement, which differs fundamentally from our focus on reconstruction in Zone IV. Regarding material properties, similar to titanium, tantalum has also emerged as a critical material for prosthetic fabrication. Houdek et al. [31] reported a study on pelvic reconstruction after resection of chondrosarcoma using customized tantalum prostheses. In their cohort treated with tantalum-based reconstruction (*n* = 10), the mean MSTS 93 score was 78% ± 23%, with a complication rate of approximately 50%. These outcomes are largely consistent with our findings (79.5% ± 13.5%). While their study did not include patients with Zone IV involvement, it is noteworthy that their follow-up period extended to 10 years, substantially longer than the short-term follow-up in our series. In terms of prognosis, as our study primarily focused on early efficacy and technology-related complications, survival analysis only explores the early follow-up results of PFS. Although it is not possible to further analyze OS due to small sample size and short-term follow-up, we have conducted a literature review beyond the scope of pelvic malignant tumors, covering all studies on the application of 3D-printed pelvic prosthesis in the treatment of pelvic tumors, combining the prognosis of its clinical characteristics, functional results, potential complications and long-term follow-up, in order to help readers comprehensively evaluate the efficacy of 3D-printed pelvic prosthesis in various disease cohorts. (Table 5).

**Table 5:** Literature review on 3D-printed pelvic prostheses for the treatment of pelvic tumors

Researcher	Parients (*n*)	Tumor type *n*(%)	Tumor zoning *n*(%)	Function	Complications *n*(%)	Survival *n*(%)
Liang et al. [10]	35	osteosarcoma: 11(31.4%); chondrosarcoma: 9(25.7%); ewing sarcoma: 6(17.2%); benign/borderline tumor: 4(11.4%); soft tissue sarcoma: 3(8.6%); metastatic carcinoma: 2(5.7%);	I: 3(8.6%); II + III: 12(34.3%); I + II: 5(14.3%); I + II + III: 2(5.7%); I + II + IV: 10 (28.5%); I + II + III + IV: 3(8.6%);	MSTS 93: ilium prosthesis(average 22.7) standard prosthesis(average 19.8) ilium-screw connection prosthesis(average 17.7)	delayed wound healing: 7(20.0%); dislocation of hip joint: 2(5.7%);	follow-up:6–30 months. DFS: 25(71.4%); AWD: 5(14.3%); DOD: 5(14.3%);
Feng et al. [13]	10	giant cell tumor of bone: 1(10.0%); soft tissue sarcoma: 3(30.0%); metastatic carcinoma: 3(30.0%); plasmacytoma: 1(10.0%); benign tumor: 1(10.0%); chondrosarcoma: 1(10.0%);	I: 2(20.0%); II: 2(20.0%); I + II: 3(30.0%) II + III: 2(20.0%); I + II + III: 1(10.0%)	VAS: 2.2 ± 0.9 MSTS 93: 19.4 ± 5.9	dislocation of joint: 2(20.0%); guillain-barre syndrome: myasthenia: 1(10.0%); delayed wound healing: 3(30.0%); wound infection: 2(20.0%);	follow-up:8–28 months. recurrence: 1(10.0%); metastasis: 4(40.0%); death: 1(10.0%);
Broekhuis et al. [14]	15	neurofibroma: 1(6.7%); osteosarcoma:3(20.0%); soft tissue sarcoma:3(20.0%); ewing sarcoma: 2(13.3%); metastatic carcinoma: 5(33.3%); giant cell tumor of bone: 1(6.7%);	Contains benign or malignant tumors in zone II.	MSTS 93: 63.3% REST STATE NRS: 0 ACTIVE STATE NRS:2.0 HOOS-PS: 76.6%	prosthesis specific complications: 4(26.7%); dislocation of hip joint: 1(6.7%); structural complication: 3(20.0%); deep infection: 1(6.7%);	follow-up:24–78.1.1 months. DFS: 11(73.3%); DOD4: 4(26.7%); recurrence: 1(6.7%);
Xu et al. [15]	10	giant cell tumor of bone: 5(50.0%); soft tissue sarcoma: 5(50.0%);	I: 5(50.0%); II: 5(50.0%);	MSTS 93: 23.8 ± 1.3	infection of incisional wound:1(10.0%)	follow-up:6–24 months.
Lv et al. [16]	6	osteosarcoma: 2 (33.3%); soft tissue sarcoma: 2(33.3%); malignant fibrous histiotoma: 1(16.7%); giant cell tumor of bone: 1(16.7%);	I + IV: 6(100.00%)	VAS: 1.33 MSTS 93: 25.33	delayed wound healing: 2(33.3%);	follow-up:18–75 months. DFS: 4(66.7%); AWD: 2(33.3%);
Dong et al. [17]	10	osteosarcoma: 3(30.0%); ewing sarcoma: 3(30.0%); metastatic carcinoma: 1(10.0%); soft tissue sarcoma: 2(20.0%); giant cell tumor of bone: 1(10.0%);	-	MSTS: 21.5	infection: 3(30.0%); poor wound healing: 1(10.0%); aseptic loosening: 1(10.0%);	follow-up:4.4–37.8 months. DFS: 6 (60.0%); DOD: 4(40.0%);
Peng et al. [18]	6	destruction of bone: 1(16.7%); soft tissue sarcoma: 5(83.3%);	I + II: 2(33.3%); II + III: 1(16.7%); I + II + III: 3(50.0%);	VAS: 2.83 ± 1.47 Harris: 58.17 ± 13.92 MSTS 93: 19.83 ± 4.26	None.	follow-up:18–42 months. DFS: 6(100.0%);
Valente et al. [19]	6 (Another 8 cases were not included in the statistics due to complications)	ewing sarcoma: 3(50.00%); osteoblastoma: 1(16.7%); giant cell tumor of bone: 1(16.7%); osteosarcoma: 1(16.6%);	-	Harris Hip Score : 83.5 ± 8.7 Barthel Index: 100;MSTS 93༚ 26.8 ± 1.9	prostheses were removed: 8 (57.1%); infection:1 (7.1%);	follow-up:12–54 months. local recurrence: 2(14.3%); prefollow-up death: 5(35.7%);
Li et al. [20]	7	They are all giant cell tumors of bone.(100.0%)	I: 3(42.8%) I + II: 1(14.3%) II + III: 2(28.6%) I + II + III: 1(14.3%)	MSTS 93: 24.4	delayed wound healing(number is not available)	follow-up : 28-45months.
Liu et al. [21]	13	soft tissue sarcoma: 5 (38.4%); giant cell tumor of bone: 1(7.7%); malignant giant cell tumor of bone: 1(7.7%); melanoma: 1(7.7%); osteosarcoma: 1(7.7%); ewing sarcoma: 2(15.4%); metastatic carcinoma: 2(15.4%);	II + III: 3(23.1%) I + II + IV: 6(46.1%) I + II: 1(7.7%) II: 2(15.4%) II + IV: 1(7.7%)	MSTS 93: 21.46	delayed wound healing: 2(15.4%); postoperative neuralgia: 2(15.4%); postoperative infection: 2(15.4%); gait of the middle gluteal muscle: 1(7.7%); internal fixation loosening: 4(30.8%); postoperative dislocation: 1(7.7%);	follow-up:1.33–31.16 months. local recurrence/metastasis: 4(30.8%);
Carmine et al. [22]	14	soft tissue sarcoma: 11(78.6%); ewing sarcoma: 2(14.3%); metastatic epithelioid angiosarcoma: 1(7.1%);	II + III: 9(64.3%) I + II + IV: 3(21.4%) I + II + III + IV: 2(14.3%)	MSTS 93: 46.3%	wound dehiscence, deep vein thrombosis, peroneal nerve paralysis, unsatisfactory reconstruction: 8(57.1%); deepeep infection: 1;rupture of illium vein: 1(7.1%); Loosening of symphysis pubis and osteolysis: 2 (14.3%); ischium loosening and screw fracture: 1(7.1%);	follow-up:15–67 months. local recurrence: 1(7.1%); 5y-OS: 62.9% metastasis: 2(14.3%); 5y-DMFS: 75.0% 5y-LRFS: 85.7%
Wu et al. [23]	28	soft tissue sarcoma: 7(25.0%); giant cell tumor of bone: 6(21.4%); osteosarcoma: 5(17.9%); malignant fibrous histiotoma: 5(17.9%); diffuse giant cell tumor of tendon sheath: 2(7.1%); metastatic carcinoma: 2(7.1%); ewing sarcoma: 1(3.6%);	I + II: 10(35.7%) I + II + III: 6(21.4%) II + III: 4(14.3%) II: 4(14.3%) I: 4(14.3%)	MSTS 93: 23.3	superficial infection: 6(21.4%); dislocation of hip join: 3(10.7%);	follow-up :3 DFS: 16(57.1%); AWD: 3(10.7%); DOD: 9(32.1%);

*DFS* disease-free survival, *AWD* alive with disease, *DOD* died of disease, *HOOS-PS* Hip disability and Osteoarthritis Outcome ScorePhysical Function Short Form, *5y-LRFS* 5-year Local Recurrence-Free Survival, *5y-DMFS* 5-year Distant Metastasis-Free Survival, *5y-OS* 5-year Overall Survival

A distinctive feature of this study lies in its rigorous analysis of early outcomes and comprehensive documentation of technical details. Furthermore, given the scarcity of existing research addressing sacroiliac joint changes following prosthetic implantation, we propose a hypothesis of potential instability risk based on imaging observations. However, it must be emphasized that this remains a speculative mechanism, and further validation through larger-sample long-term follow-up studies or computer-simulated biomechanical testing of the pelvis is required to substantiate this hypothesis. Such potential instability may represent a critical long-term concern for 3D-printed prostheses as a reconstruction method. Current knowledge gaps mainly lie in the limited assessment tools and insufficient sample data. With the ongoing advancement of artificial intelligence (AI), we anticipate that within the next five years, incorporating more variables into AI-driven simulations may enable a more objective analysis of pelvic biomechanical stability. Similarly, the integration of AI in the field of 3D-printed prosthetics could facilitate the design of perfectly matched implants. Therefore, the revolutionary progress in AI and its convergence with medical science are expected to open new avenues and breakthroughs in this research area.

This study has several limitations that should be considered when interpreting the results. First, the single-center design and small cohort size inherently limit the statistical power and generalizability of the findings. However, this initial cohort size is not uncommon in exploratory studies of this nature, given the complexity and highly customized specifics of the implant technology. Second, the retrospective design may introduce unmeasured confounding factors, although we sought to minimize bias through standardized data extraction and blinded radiological assessment. Third, the mean follow-up duration of 10.6 months, while sufficient for assessing early complications and initial integration, is undoubtedly insufficient to evaluate the long-term durability, late mechanical failure, or aseptic loosening of the custom implants, underscoring the necessity for continued future surveillance. Furthermore, the absence of a direct comparison group against alternative techniques means that definitive conclusions regarding relative advantages cannot be drawn; Finally, this study included patients with both primary and secondary malignant tumors, with the primary aim of evaluating the universal technical protocol and early-term safety of 3D-printed custom prostheses for reconstructing complex pelvic defects. All enrolled patients shared the following criteria: (1) requirement of type II or greater pelvic resection; (2) comparable biomechanical reconstruction needs; and (3) consistent postoperative functional assessment protocols. Although tumor biological behavior varied across cases, the primary endpoints of this study were prosthesis-related complications and functional recovery, rather than oncological outcomes.

## Conclusion

The use of 3D-printed custom titanium alloy prostheses for reconstructing pelvic bone defects following tumor resection leads to early pain alleviation, improved lower extremity function, favorable quality of life, and satisfactory prognostic outcomes, while also contributing to favorable stability and continuity of the reconstructed sacroiliac joint. Nonetheless, a hypothesis has been proposed suggesting potential instability risks in the contralateral unaffected sacroiliac joint, which necessitates further evaluation through biomechanical testing in larger patient cohorts.

## Supplementary Information

Supplementary Material 1.

## Data Availability

The original contributions presented in the study are included in the article/Supplementary Material，further inquiries can be directed to the corresponding author.
